# Plasticity in neuromagnetic cortical responses suggests enhanced auditory object representation

**DOI:** 10.1186/1471-2202-14-151

**Published:** 2013-12-05

**Authors:** Bernhard Ross, Shahab Jamali, Kelly L Tremblay

**Affiliations:** 1 Rotman Research Institute, Baycrest Centre, 3560 Bathurst Street, Toronto M6A 2E1, ON, Canada; 2 Department of Medical Biophysics, University of Toronto, Toronto, ON, Canada; 3 Department of Speech and Hearing Sciences, University of Washington, Seattle, Washington, USA

**Keywords:** Neural plasticity, Perceptual learning, Auditory object representation, Auditory cortex, Auditory evoked response, Magnetoencephalography

## Abstract

**Background:**

Auditory perceptual learning persistently modifies neural networks in the central nervous system. Central auditory processing comprises a hierarchy of sound analysis and integration, which transforms an acoustical signal into a meaningful object for perception. Based on latencies and source locations of auditory evoked responses, we investigated which stage of central processing undergoes neuroplastic changes when gaining auditory experience during passive listening and active perceptual training. Young healthy volunteers participated in a five-day training program to identify two pre-voiced versions of the stop-consonant syllable ‘ba’, which is an unusual speech sound to English listeners. Magnetoencephalographic (MEG) brain responses were recorded during two pre-training and one post-training sessions. Underlying cortical sources were localized, and the temporal dynamics of auditory evoked responses were analyzed.

**Results:**

After both passive listening and active training, the amplitude of the P2m wave with latency of 200 ms increased considerably. By this latency, the integration of stimulus features into an auditory object for further conscious perception is considered to be complete. Therefore the P2m changes were discussed in the light of auditory object representation. Moreover, P2m sources were localized in anterior auditory association cortex, which is part of the antero-ventral pathway for object identification. The amplitude of the earlier N1m wave, which is related to processing of sensory information, did not change over the time course of the study.

**Conclusion:**

The P2m amplitude increase and its persistence over time constitute a neuroplastic change. The P2m gain likely reflects enhanced object representation after stimulus experience and training, which enables listeners to improve their ability for scrutinizing fine differences in pre-voicing time. Different trajectories of brain and behaviour changes suggest that the preceding effect of a P2m increase relates to brain processes, which are necessary precursors of perceptual learning. Cautious discussion is required when interpreting the finding of a P2 amplitude increase between recordings before and after training and learning.

## Background

Experience-related modification of brain function constitutes a biological foundation for learning and memory [1]. In the auditory system plastic reorganization was first reported for primary sensory maps [2-4]. More recently this notion has been extended by research demonstrating perceptual learning without noticeable changes in primary sensory systems [5]. Whereas it was initially thought that cortical sensory maps are static, a current opinion is that they are continuously changing in a use dependent manner [6,7]. Although this concept of plasticity seems to apply to any level of brain processing, neurophysiological evidence exists mostly for primary sensory areas. Functional neuroimaging can help to define what neural mechanisms are involved during training and how learning modulates brain function.

The hypothesis that auditory learning affects early processing of sensory information has been supported by the findings that perceptual learning is often specific to the learned stimulus and rarely generalizes to other stimuli [8] or generalization is often incomplete [9]. As an example, early sensory processing occurs in separated frequency bands [10]. However, when training temporal aspects of sound, like duration discrimination, improved performance can generalize across different sound types and frequencies [11,12]. Collectively, these results support the hypothesis that plastic reorganization as well can take place beyond primary auditory representation at a level, where auditory sensory information is available across frequency bands. This point was reinforced by the fact that performance improvement from learning to identify speech sounds can transfer to stimuli that are phonetically closely related to the learned ones [13,14]. Different stages of brain processes are involved in auditory learning and a focus of the present study is to identify neural markers that can distinguish between early sensory processing as may be indicated by P1 and N1 waves of the auditory evoked responses and later higher order auditory processing, indicated by P2 and later responses.

Multiple stages of learning have been identified for pitch discrimination and are presumed to involve at least a bottom-up process of enhanced stimulus representation and a top-down process of improved stimulus selection [15]. This schema likely applies to perceptual learning of speech sounds, which also involves multiple levels of acoustic, phonetic, as well as linguistic analyses. Whereas different speech items may be phonologically different in terms of low-level physical sound representation, acoustic representation and differentiation alone do not directly translate into perception [16,17]. Conscious perception and comprehension of each spoken word or syllable requires high-level representation as well. For example, the identification of stop-consonant syllables differing in voice onset time (VOT) involves multiple strategies so that versions of the same syllable that are acoustically different, and spoken by different speakers, are recognized and categorized in the same way. Then again, acoustic variations impacting VOT may affect syllable identification and can change the meaning of a word. The interaction between learning at the level of the acoustics and the level of identification motivates us to question, if during auditory VOT training we are improving the distinction of the fine acoustical details, or we are building up an enhanced representation of the new items at higher level. It seems important to know about this difference for example for designing most efficient training programs. Studying the brain responses related to learning may help to answer these questions.

Neuroplastic reorganization often manifests itself as an increase in amplitude of the auditory evoked response to the learned stimulus and has been studied using electroencephalography (EEG) [18] and magnetoencephalography (MEG) [19-21]. In several studies, the amplitude of the P2 wave with latency of about 180–200 ms increased over the time course of days after training [22,23] and such amplitude increase persisted with a considerably longer time constant of retention compared to the duration of training [24]. In contrast, training induced increases in the N1 wave at about 100 ms latency have been related to life-long training in professional musicians [25,26]. Effects of expertise on the amplitude of the P2 response have been reported for more complex stimuli. For example, professional musicians showed a P2 increase for the sound of their own instrument but not for pure tones [27,28]. It has been suggested that the N1 response is generated in frequency selective tonotopically organized areas of the auditory cortex that are specific for pure tones or harmonic complexes [29], whereas the P2 response is more sensitive to complex stimuli and could indicate a processing hierarchy from simple to complex sounds. Such distinctions suggest different functional roles for N1 and P2 waves though they are still poorly understood.

Despite an increasing number of reports about neuroplastic modulation of auditory evoked responses, the functional significance of training effects on the P2 response is widely unknown. Historically, the N1 and P2 waves have been seen as a single response with biphasic morphology. Thus, in early ERP studies the response amplitude has been measured as the difference between the negative peak of the N1 and the positive peak of the P2 wave. However, several studies found that N1 and P2 amplitudes depended differentially on variation of experimental parameters [30-32]. Recently, Crowley and Colrain [33] examined different scalp topographies, effects of brain lesions, and the effects of age, sleep, and attention on the amplitudes of the N1 and P2 waves and concluded functional independence of both responses. A hint about the functional meaning of the P2 wave comes from its latency at 200 ms. Jääskeläinen [34] suggested that the earlier N1 component may serve as a gating mechanism that transfers incoming sensory information to further analysis of the auditory object in more anterior region. There is also evidence that the N1 reflects the sensory coding of stimulus onset as well as acoustic changes contained within an ongoing sound (e.g., VOT) [35,36]. At 200 ms early sensory processing has been completed and an auditory object is established in the auditory system [37].

In this study, we examined neuroplastic modulation of brain activity when participants learned to identify two versions of the stop consonant syllable ‘ba’. Our hypothesis was that improved stimulus representation and decision-making would be reflected in better stimulus identification. Moreover, the time courses of neuromagnetic brain activity should inform about the level of auditory processing, at which learning changes the stimulus representation. We therefore manipulated the pre-voicing time, which is a fine detail of the physical stimulus, and expected an increase in early sensory responses if learning was mainly specific to the physical stimulus change. However, if the stimulus representation is facilitated at the higher level of an auditory object, we would expect neuroplastic changes in later response components. Moreover, the time courses of behavioural and brain changes may explain how much training time and effort is required for each step of the learning process. Again, this knowledge is important for further optimization of learning regimen and understanding deficits in perceptual learning.

We reported previously that the amplitude of the P2m, the neuromagnetic counterpart of the EEG recorded auditory evoked response, increased substantially between the MEG recordings on subsequent days although no explicit training was performed [38]. This result was consistent with an earlier report that mere exposure to the stimulus was sufficient to induce a sustained P2 increase [39]. Here, we report neuromagnetic responses obtained before and after perceptual training and compare the effects of training with the effects of stimulus experience during pre-training sessions. Specifically we discuss the results in light of the putative role of stimulus experience during passive listening for learning.

## Results

### Performance increase during training

**Figure 1:**
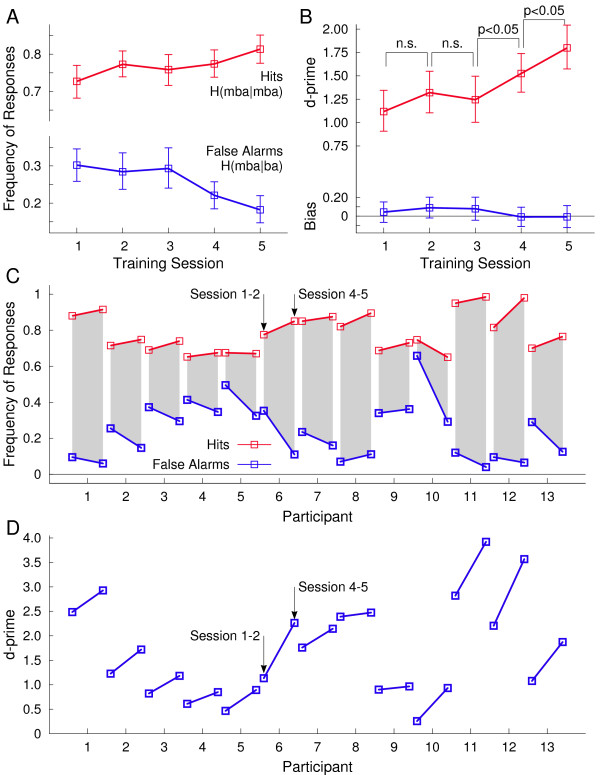
**Behavioural performance in stimulus identification during the five training sessions. A:** Labeling the speech sound with 20 ms pre-voicing time as ‘mba’ was considered as correct response. The group mean of correct responses increased over training sessions (red line). The group mean frequency of mistakenly labeling the 10-ms pre-voicing sound as ‘mba’ decreased during the training (blue line). (Error bars denote the 95%-confidence limits for the mean). **B:** The signal discrimination index d-prime (red line) increased significantly between the third and fourth and between the fourth and fifth training session, whereas the performance increase was not significant during the first half of the training. A response bias (blue) was not significant (blue line). **C:** Individual change in correct responses (red) and false alarms (blue) between the first and last training sessions. **D:** Individual changes in the signal discrimination index d-prime. Despite individual variability the d-prime measure increased for all participants during the training.

Participants were trained to identify the speech sound with the longer pre-voicing time as ‘mba’ and the one with shorter pre-voicing as ‘ba’. During the five days of training, correctly identifying ‘mba’ (a hit) increased across the group from 72.7% to 81.3% while mistakenly labeling ‘ba’ as ‘mba’ (a false alarm) decreased from 30.2% to 18.2% (Figure 1A). Correspondingly, the d’-measure increased from 1.12 to 1.80 (F(1,13) = 8.29, p = 0.013). A response bias was not significant in any training session (Figure 1B). Although the amount of increase in the hit rate of was variable across participants (Figure 1C) all individuals improved their performance as indicated by the d’-measure (Figure 1D). Identification performance did not increase significantly within the first three training sessions but improved between the third and fourth and between the fourth and fifth session (p < 0.05 both). This means that behavioural accounts of learning occurred relatively late after an accumulation of stimulus experience, beyond the initial intervals that could have involved procedural learning.

### Cortical sources of auditory evoked responses

**Figure 2:**
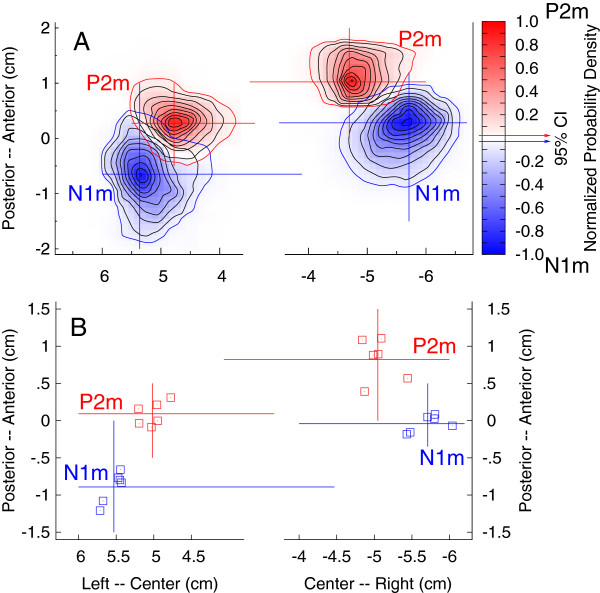
**Locations of N1m and P2m dipole sources in the axial plane. A:** Two dimensional probability density functions for dipole locations found with bootstrap resampling for the ‘mba’ stimulus during the first pre-training session. The blue and red contour lines indicate the 95% confidence limits for N1m and P2m source localizations, respectively. P2m sources are more anteriorly and medially located compared to N1m sources and right hemispheric sources are more anteriorly located than left hemispheric sources. **B:** Group mean N1m and P2m source locations observed for two speech stimuli and three sessions indicate consistently separated N1m and P2m sources.

Brain areas activated by the auditory stimuli could be identified in all participants. Locations of equivalent current dipoles for the P2m response were found in bilateral temporal lobes with center of gravity overlapping the anterior lateral parts of Heschl’s gyrus. Details of the source analysis have been reported previously [38]. To emphasize the relative locations of N1m and P2m sources in the axial plane, two-dimensional probability density functions for the source locations, obtained from bootstrap resampling, are shown in Figure 2A for the responses to the ‘mba’ stimulus in the first pre-training session. For both hemispheres, the 95% confidence limits of the P2m source did not include the mean N1m location and vice versa, indicating significant separation of N1m and P2m dipole sources. P2m sources were more anteriorly and medially located than N1m sources and right hemispheric sources were located anterior to the left hemispheric sources. The group mean N1m and P2m locations in the axial plane obtained from 12 repeated MEG recordings (two stimuli, three sessions, and two repetitions, Figure 2B) showed consistently more anteriorly located P2m sources (right: 10.0 mm, t(17) = 12.10, p < 0.0001, left: 5.2 mm, t(17) = 6.90, p < 0.0001) and a larger separation in the right hemisphere (t(17) = 3.55, p = 0.0025). P2m sources were located more medial than those of N1m (right: 5.2 mm, t(17) = 6.9, p < 0.0001, left: 3.0 mm, t(17) = 2.79, p = 0.0125).

### Training and experience induced changes in cortical responses

**Figure 3:**
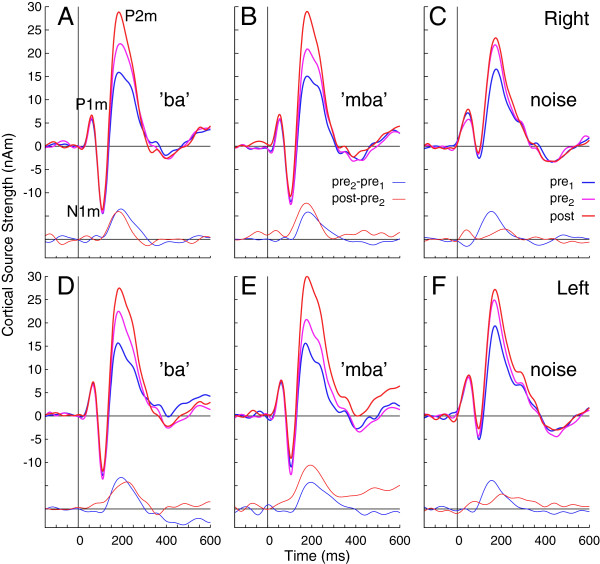
**Time series of auditory cortex activation by the speech and noise stimuli.** The six panels show separately the responses observed for the three stimuli and the right and left auditory cortex. The waveforms recorded during the three MEG sessions are overlaid and reveal a steady increase of the amplitude of the P2m wave between sessions. At the bottom of each panel additional time series of the differences between sessions are shown, indicating the amplitude change between both pre-training sessions (blue) and between the second pre-training and the post-training session (red).

Waveforms of cortical source activity, i.e. the dipole moments of the underlying equivalent current dipoles, were estimated based on the P2m source model for all subjects. Characteristic P1m, N1m, and P2m waves of the auditory evoked response were clearly expressed in the grand averaged source waveforms obtained in left and right auditory cortex (Figure 3). Inspection of the overlaid response waveforms obtained at different days provides the general schema that largest changes between sessions occurred around the P2m latency, while the P1m and N1m waves had almost identical amplitudes across all sessions. The P2m amplitude increased between the pre-training sessions and between pre- and post-training sessions. The amount of increase was of similar size for the speech stimuli, but was smaller for the noise stimuli after the pre-training sessions as indicated by the difference time series in Figure 3.

**Figure 4:**
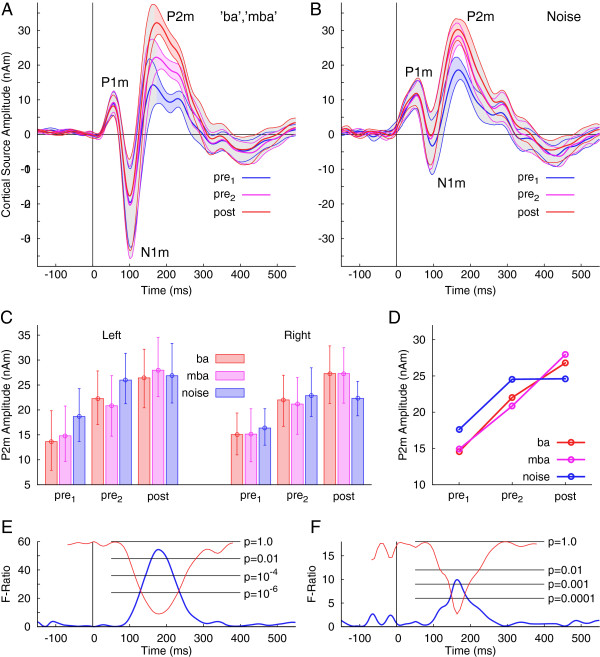
**Group statistics on the source waveforms. A:** Group mean responses (thick lines) to the speech stimuli during the three MEG recording sessions and the 95% confidence limits (thin lines) for the group mean as estimated by bootstrap resampling. The confidence bands for the three days of MEG recording overlap widely, however, they are separated for all sessions in a latency interval around 200 ms (colored shaded areas), indicating significant differences in the group mean amplitudes during the P2m latency interval. **B:** Group mean responses and 95% confidence limits for the responses to the noise stimulus. The confidence bands do not overlap during the P2m latency interval for the first and second pre-training sessions only. **C:** Group mean P2m amplitudes for all experimental conditions. The between subject variability is illustrated with the error bars, depicting the 95% confidence intervals for the mean. **D:** Mean P2m amplitudes for the three stimuli and the three sessions illustrate the interaction between stimulus and session. **E:** Main effects of the recording session as revealed by repeated measures ANOVA calculated for each time point separately. The F-ratio took high values around 200 ms exclusively correspondingly to high significance for the effect of the recording session on the response amplitude. **F:** The time course of the interaction between session and stimulus type shows significance during a latency interval around 200 ms only.

Confidence limits for the group averages, estimated from bootstrap resampling for the different sessions, overlapped specifically for the P1m and N1m waves (Figure 4A). Confidence bands were non-overlapping between sessions for a short latency interval around 200 ms for the speech stimuli. For the noise stimulus, the confidence intervals for the second pre-training and the post-training session overlapped almost completely (Figure 4B), indicating no further P2m increase after the second pre-training MEG recording.

The P2m amplitude, defined as the mean amplitude in the latency interval from 180 to 220 ms, was analyzed by a repeated measures ANOVA with the within group factors *stimulus* (three levels: ‘ba’ , ‘mba’ , noise), *session* (three levels: pre_1_, pre_2_, post), and *hemisphere* (left and right). The mean amplitude measures are summarized with a bar diagram in Figure 4C. The ANOVA revealed a main effect of the factor *session* (F(2,24) = 33.6, p < 0.0001) because the mean P2m amplitude increased between the pre-training sessions by 43% (t(12) = 4.8, p = 0.0003) and between the second pre- and post-training sessions by 18% (t(12) = 3.4, p < 0.0053). In total the P2m amplitude increased by 69% of its pre-training value. The mean P2m amplitudes were not different between right (21 nAm) and left (22 nAm) hemispheres (F(1,12) <0.2). A *session* x *stimulus* interaction (F(4,48) = 11.7, p < 0.0001) was significant because the P2m amplitude for the noise did not increase between pre- and post-training sessions (Figure 4D). The P2m amplitude for the noise stimulus increased by 40% between the pre-training sessions (t(12) = 6.0, p < 0.0001), but did not increase between pre- and post-training sessions (t(12) < 0.1, n.s.). In contrast, for the speech stimuli, the absolute amplitude increase by 6.7 nAm between pre-training sessions was not different from the increase by 5.9 nAm between second pre-training and post-training sessions (t(12) = 0.35, n.s.).

The ANOVA performed for each time point revealed that the main effect of the factor *session* and the interaction between *session* and *stimulus* were specific for the latency interval around 200 ms. The time courses of the F-ratio and the corresponding p-value showed only a single significant peak close to 200 ms (Figure 4E, F).

### Spatio-temporal source imaging

Multiple components of the N1 response are generated in the lateral part of Heschl’s gyrus and the planum temporale [40,41]. According to the relative distances found between N1m and P2m sources, we assumed sources of the P2m to be located in anterior auditory cortices and discussed its functional meaning based on current opinions about auditory processing in this area.

**Figure 5:**
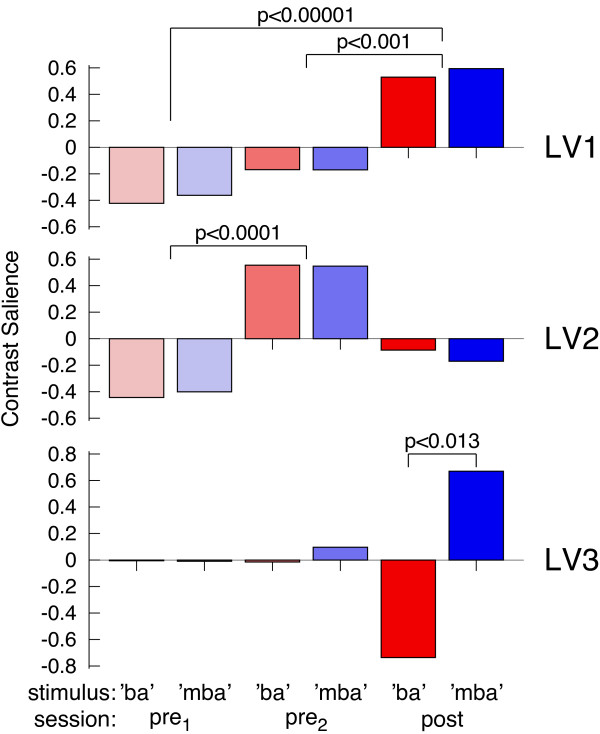
**Three largest latent variables resulting from multivariate analysis of auditory evoked source activity.** The first latent variable LV1 steadily increases between MEG recording sessions, LV2 shows a contrast between the two pre-training sessions, and LV3 shows a contrast between ‘ba’ and ‘mba’ after the training only.

**Figure 6:**
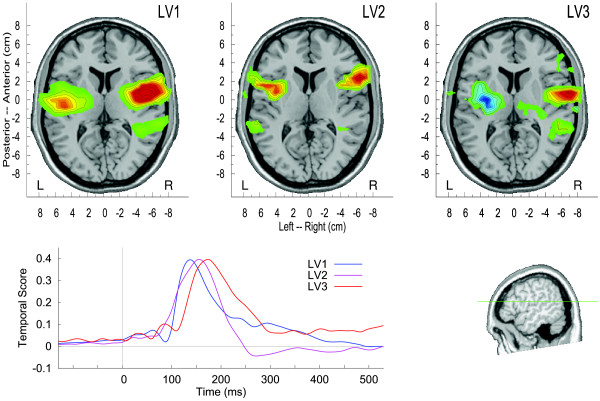
**Spatio-temporal brain activation patterns corresponding to the three largest latent variables.** The amplitudes of the waveforms and the scale of the activation maps are normalized. The latent variables contributed differentially to the effects of the experimental parameters, LV1 explained 87% of the variance, LV2 12%, and LV3 8%.

Modeling the brain activity in bilateral temporal lobes with single equivalent dipoles was effective for investigating the overall effects of *sessions* and *stimulus* types on the response amplitude. For studying a possible differentiation in the responses to the trained stimuli we used a whole brain source imaging approach and applied multivariate partial least squares analysis on the spatio-temporal maps of the auditory evoked response. This entirely data driven approach decomposed the brain activity into factors, which were related by latent variables (LV) to the experimental conditions. How the three largest LVs contributed to explain the data is illustrated in Figure 5. The first LV related to a monotonous change in source activity between both pre-training sessions and between pre- and post-training MEG sessions. This factor was predominant and LV1 explained 67% of the variance in the data. The second factor showed a contrast specific for the pre-training sessions, not involving the change between pre- and post-training sessions and explained 12% of the variance. The third factor, explaining 8% of the variance, showed a contrast between the responses to ‘ba’ and ‘mba’ , which was evident after the training only. The corresponding time courses and spatial maps are shown in Figure 6. The time courses demonstrate that all factors were concentrated around the P2 latency interval. Although the peak latencies of the latent variables in the 150 ms to 180 ms range seems to appear earlier than the peak latency of the P2 wave at 200 ms. This can be explained with the specific sensitivity of the PLS to fine latency differences [42]. Thus, the LVs showed a peak in the latency range of largest P2 change during P2 onset, which sometimes even overlaps with the N1 latency range. The spatial map corresponding to LV1 shows centers of activity anterior to Heschl’s gyrus and the activity related to LV2 was located even more anteriorly. Whereas the effects indicated by LV1 and LV2 were bilaterally organized, LV3 was lateralized toward the right hemisphere.

## Discussion

The main findings were that focused listening during perceptual training, as well as passive stimulus experience during MEG recording, constituted sustained increases in evoked activity in anterior auditory cortex 200 ms after stimulus onset. The amplitude gain between pre- and post-training MEG recording was larger for the speech stimuli than for the noise stimulus, which was not involved in the training and participants were less exposed to. Amplitudes of the P1m and N1m responses, which have shorter latencies, were not significantly modulated. Multivariate analysis identified three distinct spatio-temporal patterns of brain activity related to the increase in P2m across the three recording sessions, changes between pre-training sessions, as well as differences between the responses to the trained stimuli, which were evident after training only. Each result is addressed below.

### Trajectory of behavioural performance

All individuals improved in their ability to identify the stimuli over the time course of the study. Notably, the improvement became evident during the last days of training, whereas the group-mean performance was not significantly different between the first days. This trajectory of learning during the early part of training is different from the time course of early improvement in pitch discrimination learning for example. For changes in pitch discrimination performance, it has been reported that the strongest gain occurred at the beginning and performance reached a maximum asymptotically with smallest gain at the end of training [43,44]. Similar time courses showing large initial improvement have been found when learning discrimination of interaural time and level differences [45]. In contrast, the behavioural improvement in this study occurred during the later sessions. It seems that for the stimulus identification task in this study, the participants first had to establish and adjust a categorical boundary, and the effect of training translated later into a behavioural consequence. Thus, we speculate that some implicit learning took place at the early stage of the training procedure.

### Trajectory of brain responses

Previous analysis of N1m and P2m amplitudes showed a significant difference in the trajectories of changes in the N1m response and the P2m response. The P2m amplitude was constant during a recording session while it increased between the end of the first and beginning of the second pre-training session on a later day. Consolidation during a night of sleep seems to be important for this P2m gain. In contrast, the N1m amplitude decreased within a session but recovered between sessions [38]. Recent studies corroborated our observations about the time course of P2m changes [46]. The finding that changes in P2m amplitude occurred with a delay of one or more days is consistent with reports of Atienza et al. [23,47] that the amplitudes of P2 and mismatch negativity (MMN) responses increased over days after training but not during the EEG recordings. P2 amplitude changes were also not seen when training involved easier VOT contrasts in a brief single session recording [48]. Collectively, these examples reinforce the notion that N1 and P2 reflect different neural sources that are differentially affected by time and task. Therefore we propose that the gain in P2m amplitude between sessions reflects the cumulative effect of passive and active listening during the time interval from beginning of the first to beginning of the second session. Moreover, a change in the P2m amplitude between the two pre-training recordings reflects the effect of stimulus experience during the first session but is not affected by sound exposure during the second session. Accordingly, the amplitude change between the second pre-training and the post-training sessions includes the effects of passive listening to the stimuli during the pre-training session and active listening during the training. Only the P2m amplitude recorded in the first session can serve as an estimate of a pre-experimental baseline.

According to our previous studies, when P2 amplitudes were seen across multiple days of stimulus experience, the retention of these P2 changes was surprisingly long lasting compared to the time interval of acquisition [24]. For example, even one year after the first recording, the P2 amplitude exceeded the initial amplitude. We interpreted this type of response increase to be a part of learning in that repeatedly presented sounds become familiar [38] and contribute to the enhanced representation of the implicitly learned stimulus, but fall below the threshold of learning that has a behavioural consequence [49]. Thus, a certain amount of stimulus experience without performing a specific task seems to contribute to perceptual learning. This point is reinforced by results from a frequency discrimination experiment where participants improved their ability to discriminate identical stimuli through focused listening training. Repeated exposure and focused attention resulted in perceptual gains, even though discrimination was not possible because the stimuli were identical [15].

### P2m change in relation to the stimulus type

A gain in the P2 amplitude has been reported in several training studies involving different stimuli and tasks, thus the P2 gain is not unique to identification of a pre-voicing interval. In this study the P2m amplitude, elicited by the noise stimulus, increased between the pre-training sessions by a similar amount as the P2m amplitude gain for the speech stimuli. However, further P2m increase between the second pre-training and the post-training sessions was not significant for the noise stimulus. Keeping in mind that the noise stimuli were used during MEG recordings only, these data suggest that the effect of passive stimulus experience saturated after the first session. Considering the results of previous studies that the observed P2 gain is widely independent of the stimulus material, the time course of the P2 changes for the noise stimulus helps to make a reasonable assumption about the contribution of passive stimulus experience during the second MEG session to the P2 increase. The P2m response for the speech stimuli continued to increase between the second pre-training and the post-training sessions with an effect size similar to the effect of listening during the first MEG session. This P2m increment resulted from the cumulative effects of active listening during five days of identification training and passive listening during the second MEG session. Given the small increase for the noise stimulus, we take this as an estimate for the effect of stimulus experience during the second MEG session and assume that the P2m increase between the second and the third measurement can be attributed by far to the effect listening during the training. Still we do not know how the additive effects of continued stimulus experience and active auditory processing related to the perceptual task contributed to the modulation of P2m amplitude during training. It seems that different neural mechanisms contribute to the P2 increase.

Interestingly, the beamformer analysis revealed a spatio-temporal pattern of activity in bilateral anterior auditory cortices, which was specific for the change between the two pre-training sessions but was not involved in further change during the training. Further studies are required for identifying which property of the training procedure effectively induced performance increase and gain in brain responses.

### An argument for enhanced object representation

Auditory evoked P2m responses in the 200 ms latency range were strongly modulated after active and passive listening. To interpret the functional significance of P2m changes, it is important to discuss what happens in the 200 ms latency range during auditory processing. When a sound is heard, the auditory system performs a complex spectro-temporal analysis involving a hierarchy of processing steps within the auditory pathways [50-52]. Sound features like spectral complexity, frequency transitions, and rhythm are already extracted by this time and processed by nuclei in the auditory midbrain [53]. The role of the auditory cortex is to enhance such features and to organize the acoustical elements into an object [54]. Näätänen and Winkler [37] described the initial storage of sensory information as expression of feature traces. Components of the auditory evoked N1 wave reflect this stage, indicating that the auditory information is present at the level of auditory cortex, but not yet accessible for conscious perception. As an example, changes in voice onset time are evident by the time they reach auditory cortex [35,55] and are reflected in amplitude and latency of the N1 response. However perception of the VOT according to categorical boundaries that differentiate syllables depends on further processing, and is strongly influenced by experience [56]. Reaction time studies also reinforce that one or two-syllable words are accessible about 200 ms after word onset [57]. Therefore, it can be said that this 200-ms time window includes the time required for bottom up processing of acoustical information as well the time required for comparison with contextual information.

Whereas Näätänen and Winkler [37] used the term ‘stimulus representation’ in contrast to a ‘pre-representational’ stage as reflected in the N1 response, we prefer the term ‘auditory object representation’. The ‘auditory object’ was initially referred to as a construct having a visual equivalence [58], however it is now more generally used for auditory sensory information that is susceptible to figure-ground segregation and involves a level of abstraction so that information about the object can be generalized between sensory experiences even across sensory domains [59]. At 200 ms latency, the neural representation of an auditory object is established and now accessible for further conscious processing. Näätänen and Winkler [37] discussed the 200-ms activity in terms of the MMN rather than the P2, which is the difference between the response to an infrequent deviant stimulus and a more frequently presented standard stimulus, and reflects the result of comparing incoming stimuli with the memory trace established by the standard stimulus. We chose to use a different experimental approach whereby repeated presentations of the same stimuli were used to evoke a P1-N1-P2 response instead of an MMN response. The intention behind our approach was to use an evoked response (e.g., P1-N1-P2) that could more easily be defined in individuals and might one day be clinically applicable in the study of people with communication disorders. Moreover, the stimulus presentation paradigm and identification task are more similar to one another in that discriminative processes are not being activated. With that said, Atienza et al. [23,47] reported similar trajectories for plastic changes in MMN and P2 responses which supports a possible link between the two types of evoked responses and shared neural mechanisms.

### Sources in anterior auditory cortex

Although P2 source localization has been described as difficult [28,41], we found significant separation between P2m and N1m sources, which is consistent with earlier neuromagnetic findings of P2m sources located approximately 10 mm anterior and 5 mm medial to N1m [60]. Neuroimaging studies have linked auditory object representation to the anterior auditory cortex. More specifically, there is evidence of preferred firing patterns for animal calls in anterior lateral part of monkey superior temporal gyrus and the caudolateral part responding to location cues. Together they help to established a dissociation of ‘what’ and ‘where’ pathways in auditory processing [61,62]. The concept of processing the sound object in anterior and the spatial information in posterior auditory cortex has been reinforced by animal studies [63] and human studies [64,65]. Specifically, areas in the anterior superior temporal plane have been shown to be responsive for auditory objects [66].

### Specificity for the learned stimulus difference

Perceptual learning changes the way in which the trained object is represented and processed in the brain [1]. Accordingly, a difference in neural representations of the trained stimuli should emerge after the training. Using entirely data-driven multivariate analysis, we found a spatio-temporal response component that differentiated the ‘mba’ and ‘ba’ responses after training only. Moreover, this analysis demonstrated that the spatio-temporal patterns of brain activity were different for the contrast between the two pre-training sessions and between post- and pre-training. Although the P2m amplitude increased bilaterally and no main effect of hemispheres was significant in the analysis of equivalent dipoles, the difference between responses to the speech stimuli was mostly expressed in the right anterior auditory cortex. This specific activity emerged during the late part of the P2m complex, and supports our opinion about auditory object representation. In the literature, discussing hemispheric specialization, the right hemisphere has been shown to be involved in spectral processing whereas the left hemisphere predominantly processes temporal fine structures [54]. However, a specialization for fine pitch discrimination requires some integration over time and an asymmetry for integration times has been proposed with longer integration time (150–200 ms) in the right hemisphere and shorter integration time in the left hemisphere (20–40 ms) [67]. Longer integration times may facilitate object processing in the right hemisphere. Accordingly, specific sensitivity of the right anterior auditory cortex for object processing has been concluded from a PET study [68]. Moreover, in a study of detecting the direction of frequency sweeps in frequency-modulated tones in gerbils, a hemispheric asymmetry was found and was suggested to be a precursor of the organization of music and language in humans; the left auditory cortex was more involved in local processing of temporal fine structure, whereas more global processing used the right auditory cortex [69].

### Sensation versus object representation

The ‘reverse hierarchy theory’ of perception [17] proposes that spectral components of an auditory stimulus are initially separately received, then integrated during auditory processing, and the auditory object becomes accessible for perception only at a higher level of object representation. In order to distinguish between phonologically similar stimuli, the listener has to scrutinize the sounds carefully, which usually requires stimulus repetitions, to gain access to finely structured details, represented at lower level within the sensory hierarchy. The stimuli used here were spectrally identical but differed in timing. This means perceptual training could have either improved the ability to access such lower level stimulus features (such as the temporal VOT cue) or established new object representations of each stimulus. Because our results of brain activity changes in the 200-ms latency range and sources in the anterior auditory cortex support the latter, we suggest that identification training forced participants to attach a label to each stimulus, which in turn generated separated objects. This point is reinforced by the fact that our analyses suggest that each speech stimulus was represented differently after training.

Behavioural improvement was significant in the last half of the training but not within the first days. In contrast, brain changes were evident already between the pre-training sessions. Thus the trajectories of behavioural performance and brain responses were essentially different. Changes in the brain responses seem to precede behavioural performance. This again is consistent with our concept that learning first builds a strong representation of the auditory objects, which in turn allows the participant learning to identify the subtle differences between stimuli.

Based on the temporal and spatial information obtained in our study, we propose that perceptual learning and training result in plastic reorganization at the level of object representation. In contrast, we did not find significant indications for plastic changes of the P1m and N1m responses, which are both thought to signal stimulus changes at a sensory level, which would be indicative for early sensory processing of the trained subtle stimulus differences in pre-voicing time. In contrast to the absence of detectable changes in the P1m and N1m responses in our study, strong neuroplastic changes in early primary auditory responses had been found in perceptual learning in animal studies [70-73] and in human auditory evoked responses [74-76]. Common to those studies was that the spectro-temporal differences in the stimuli were larger than in our current study and perceptual learning as well as neurophysiological changes occurred rapidly. The differences between studies indicate that it is important to discuss the experimental findings always in the context of the experimental conditions.

### Potential effect of attention

Active and passive listening might have altered the way participants attended to the stimuli. Although the MEG recording was performed under passive listening conditions, that did not require directed attention, the speech stimuli may have become more salient after learning and may have captured more attention in later MEG sessions compared to the first one. The effect of attention on the auditory evoked response in the 200-ms latency range has been described in ERP recordings as a long lasting negative wave Nd [77] or processing negativity [78], both with similar scalp topography as the P2 wave. Because of increased negativity at same latency as P2 in total, the P2 amplitude decreases (rather than increases) with attention [40,79,80]. Although attention might have modulated the effect of stimulus experience and of training, it seems unlikely that changes in attention between blocks of different stimuli and between MEG recording sessions can explain the P2m amplitude increases observed in this study. The Nd wave or the processing negativity is strongest when an active task is involved. For this reason we chose to avoid such compromising effects on the P2m amplitude by using a passive listening paradigm for the MEG recording.

### The P2 response as an indicator for learning

In this training study the P2 response showed remarkable neuroplastic modulation. However, multiple stages of learning are involved, and we have to differentiate carefully between those when relating the observed P2 changes to learning and training. It seems that the P2 amplitude does not reflect a straightforward brain-behaviour relationship. Instead it seems as if the P2 amplitude indicates facilitation of implicit memory for the auditory object that precedes any perceptual change. The increased object representation is an essential part of learning and allows the listener to access details in the sensory representation, which in turn permits the correct identification of phonetically similar objects and potentially even categorical perception. Interestingly, the amplitudes of brain activity and behaviour follow different trajectories over time. The gain in P2 amplitude was delayed with respect to the time of stimulus experiences, thus suggesting effects of neural consolidation. On the other hand, the gain in P2 amplitude preceded an improvement in performance, again suggesting its role in implicit learning.

## Conclusions

We interpret our finding of plastic modulations in the 200-ms latency range as being consistent in time with neural networking involved in the recognition of an auditory object. Although training of a pre-voicing contrast for stop consonant syllables led to improvement in identifying the syllables, the behavioural improvement occurred late in the time course of training. Substantial increase in brain activity with 200 ms latency and sources in anterior auditory cortex indicated neuroplastic changes over the time course of implicit and explicit learning, which were recorded as the P2 wave of the auditory evoked response. Changes in brain activity became evident before behavioural consequences emerged. Multiple stages of learning have to be considered when attributing the changes in brain activity to learning. Stimulus experience during passive listening as well as active involvement in a stimulus identification task contributed to learning and neuroplasticity. Earlier brain activity, measured with the P1 and N1 responses, did not show neuroplastic changes, thus learning in this study was more reflected in higher-level auditory object representation rather than lower-level sensory processing. When interpreting the neuroplastic changes within the framework of object specific learning, we propose that P2m represents the automatic recognition of recently experienced auditory objects, and P2m amplitude increases, which persist over time, reflect enhanced object representation so that participants, later in time and with training, can scrutinize fine differences in pre-voicing and in turn improve perception.

## Methods

### Participants

Fifteen young right-handed adults participated in this study. The inclusion criterion was that they learned English as the only native language and were using predominantly English in daily life. Partial results for this same group have been reported in a previous publication where the effects of stimulus experience, during the pre-training sessions, were examined [38]. Two individuals did not complete the training, thus, six female and seven male (age 19–33 years, mean 25.2 years) were included in this study. All participants were in good general health and reported no history of otological or neurological disorders. They had normal hearing bilaterally, defined as audiometric pure tone thresholds better than 25 dB nHL between 250 Hz and 4000 Hz. Participants provided their written consent after receiving information about the nature of the study, which had been approved by the Research Ethics Board at Baycrest Centre as well as the University of Washington.

### Auditory stimuli

**Figure 7:**
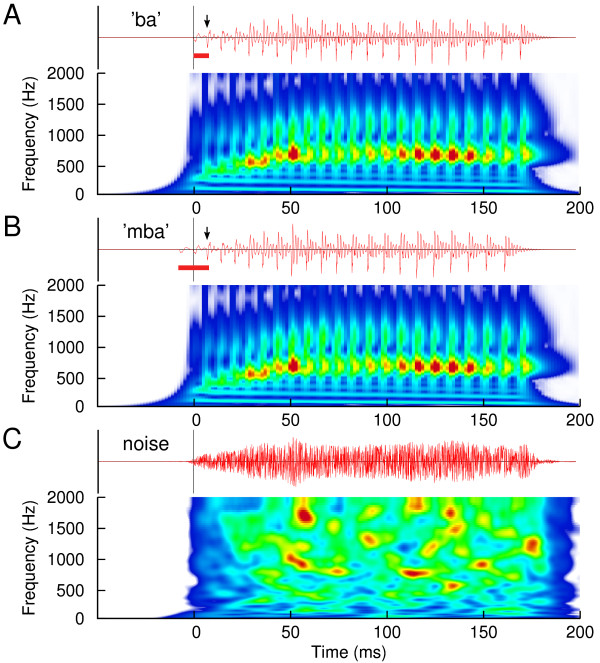
**Time series and spectrograms of the stimuli of 180 ms duration. A:** The spectrogram of the ‘ba’ sound, limited at 2000 Hz, reveals the first formant at 700 Hz, the second at 1200 Hz, and the fundamental frequency of 120 Hz at t = 0 falling to 100 Hz at the end. Time zero is adjusted to the onset of the ‘ba’ stimulus. **B:** The ‘mba’ sound differed from the ‘ba’ sound by 10 ms of additional pre-voicing. The arrows indicate the voice onset. The pre-voicing intervals of 10 ms for ‘ba’ and 20 ms for ‘mba’ are marked with red bars below the time series. **C:** Time series and spectrogram of the frozen noise stimulus.

Two versions of pre-voiced stop-consonant synthesized syllables ‘ba’ were used during the identification task. Pre-voicing values were 20 ms and 10 ms. For native English speakers, pre-voicing is not phonemic [13]. Therefore, without training, English speakers perceive both stimuli as ‘ba’. With training they can learn to identify the 20 ms pre-voiced stimulus as ‘mba’ and the 10 ms pre-voiced one as ‘ba’. These same stimuli have been used in previous studies and the technical details have been described [14,22,81,82]. Waveforms of the stimuli showing the temporal fine structure as well as spectrograms illustrating the transitions of first and second formant frequencies are shown in Figure 7A-B. The only physical difference between both stimuli was the brief interval prior to voice onset. A third stimulus was a noise sound, which was generated by multiplying Gaussian noise with the smoothed envelope of the ‘ba’ sound (Figure 7C). The noise stimulus was presented during the MEG sessions only, thus it informs us about the effects of passive listening during the MEG sessions without training. The stimuli were presented with a constant stimulus onset asynchrony (SOA) of 2175 ms. Although with a longer SOA the response amplitude may increase a longer recording time for the same number of responses would be required. When designing the SOA with the aim of obtaining the best signal-to-noise ratio in given recording time, the choice of 2175 ms is quasi optimal. Two hundred stimuli of the same type (i.e. 20 ms pre-voicing, 10 ms, or noise) were presented in a block of 435-s duration (7.25 min). The blocked design was preferred over stimulus randomization to avoid serial interactions between stimuli of different or same type in latter case. Especially we considered that the speech stimuli may have sounded more different after training and response interactions may be different in pre- and post-training recordings. In the first half of the MEG session, three blocks with the three different stimuli were presented in random order. The procedure was repeated in the second half of the MEG session again with blocks in randomly permuted order so that each participant heard a total of 400 repetitions of each stimulus. The duration of the MEG session was about 50 min.

### Experimental procedure

The entire procedure involved eight sessions on different days. MEG recordings were performed on two different days before and one day following five days of training. The two pre-training sessions (sessions 1 and 2) served as a control condition to examine the effects of mere stimulus exposure, independent of the training task, on brain and behavioral responses. Behavioral and MEG testing were conducted separately and the order of testing was counterbalanced across subjects. For some participants it was not feasible to schedule the pre-training sessions on consecutive days. One exception was a delay of 20 days. Otherwise, six were recorded on the following day and six within a week (mean 2.7 days, std. dev. 2.3 days). Stimuli were presented binaurally at 85 dB sound pressure level through Etymotic ER3A insert earphones connected with 1.5 m of plastic tubing. The training sessions were also performed with the participants seated in the MEG chair, using the same stimulation equipment was used for the MEG recording.

### Training sessions

Five training sessions followed the two pre-training sessions. The number of days between each training session was not strictly controlled for although each individual did participate in their post-training test session on the day immediately following the final training session. The time interval between the second pre-training and the post-training MEG recording was 15.6 in mean (range 7 to 22 days, std. dev. 4.6 days). At the beginning of the training, fifty easily distinguishable syllables with pre-voicing times of 30 ms versus 10 ms were introduced to the participants to familiarize them with the stimuli as well as the forced-choice task. Each training session that followed consisted of four blocks consisting of a total of 200 trials. Twenty-five stimuli with 20-ms pre-voicing, and 25 stimuli with 10-ms pre-voicing were presented in random order in each block. The task was self-paced. After each stimulus presentation, two labels ‘ba’ and ‘mba’ appeared on the computer screen and the participant indicated their choice with a mouse click. A green light appeared on the computer screen for 1 s when the participants correctly identified the stimulus with 20 ms of pre-voicing as ‘mba’ and the 10 ms stimulus as ‘ba’, a red light appeared for an incorrect response. After a 1-s delay the next stimulus was presented. After the block of 50 stimulus presentations was completed the program paused, and the participant decided when to continue.

### Data recording

MEG recordings were performed in a silent magnetically shielded room using a 151-channel whole-head MEG system (VSM-Medtech, Port Coquitlam, BC, Canada) at the Rotman Research Institute. The detection coils of this MEG device are equally spaced on the helmet shaped surface and are configured as first order axial gradiometers [83]. After low-pass filtering at 200 Hz, the magnetic field data were sampled at the rate of 625 Hz and stored continuously. MEG data were collected during passive listening. The participants were not required to attend to the stimuli or execute a task but were asked to remain alert. In order to control for confounding changes in vigilance, the subjects watched a closed captioned movie of their choice, while the auditory stimuli were presented. Compliance was verified using video monitoring, and ongoing MEG signals were inspected for alpha activity and eye movements. Participants were seated comfortably in an upright position with the head resting inside the helmet-shaped MEG sensor array. The head position was registered at the beginning and end of each recording block of 7.25 min duration using the three detection coils attached to the subject’s nasion and the pre-auricular points. The mean of the repeated head coil coordinates defined a Cartesian coordinate system with the origin at the midpoint between the bilateral preauricular points. The postero-anterior x-axis was oriented from the origin to the nasion, the medio-lateral y-axis (positive toward the left ear) was the perpendicular to x in the plane of the three fiducials, and the inferior-superior z-axis was perpendicular to the x-y plane (positive toward the vertex). A block was repeated when the fiducial locations differed more than ±4 mm from the mean. Out of a total of 234 recorded MEG blocks eight had to be repeated because of head movements. This procedure ensured that head movements did not significantly affect the source localization accuracy.

### Data analysis based on dipole modeling

Each block of continuously recorded MEG data was subdivided into 200 stimulus related epochs of 1500 ms duration including a 500 ms pre-stimulus interval. Principal component analysis was performed on each epoch and components exceeding the threshold of 2 pT in at least one channel were assumed as artifacts and subtracted from the data. This approach effectively removed large amplitude artifacts such as those caused by eye blinks [84]. After artifact correction, the data were averaged and magnetic source analysis was applied separately to the ±20 ms time intervals around the maximum of the N1m and P2m waves at about 100 and 200 ms latency relative to stimulus onset. The source analysis was based on the model of spatio-temporal equivalent current dipoles (ECD) in a spherical volume conductor. Single dipoles in left and right temporal lobes were fit simultaneously to the 151-channel magnetic field distribution. Dipole fits were accepted if the calculated fields explained at least 85% of the variance of the measured magnetic field.

Confidence limits for P2m and N1m source locations were estimated using non-parametric bootstrap resampling [85]. For each recording session and each speech stimulus, 1000 random samples with replacement were made from the group of participants and dipole fitting was applied to the grand averaged data of each sample for the N1m and P2m latency interval. Empirical probability density functions were obtained from the source coordinates for the left and right hemispheric N1m and P2m sources respectively. The median of spatial coordinates and orientations of the P2m sources was used as individual model to measure the source waveforms for the auditory evoked responses. Dipole moment waveforms were analyzed representing the source activity in the auditory cortices. The method of source space projection [86,87], was applied to combine the 151 waveforms of magnetic field strength into a single waveform of a magnetic dipole moment measured in nanoAmpere-meter (nAm). For calculating the waveforms of source activity, the position and orientation of the dipole model were kept constant for all time points. The polarity of source waveforms was adjusted so that the N1m peak at about 100 ms latency was negative according to the polarity of an EEG recording from a fronto-central electrode. The dipole moment measure is spatially selective for activity in the localized brain area and less sensitive to electro-magnetic sources at other locations. Also source waveforms may show higher signal-to-noise ratio than magnetic field waveforms [86]. A further advantage of analysis in source domain is that the dipole moment is independent of the sensor position and the waveforms of cortical source activity can then be combined across repeated sessions and participants. Bootstrap resampling was applied for estimating the 95% confidence limits for the group averages. Repeated measures ANOVA with the within group factors *stimulus type* (‘ba’ , ‘mba’ , and noise), *session* (pre_1_, pre_2_, post), and *hemisphere* (left, right) was applied to each time point of the waveforms of cortical activity. The absolute maximum in the time courses of the F-statistic indicated the latencies of main effects and interactions. Before applying the ANOVA we tested if the data were normally distributed and the variances were stationary across conditions.

### Event related beamformer source analysis

Whereas modeling with a single equivalent dipole in each hemisphere provided measures of combined activity of multiple auditory areas in each hemisphere, beamformer source imaging could potentially distinguish between components of source activity based on different spatio-temporal patterns. The beamformer source imaging approach improves spatial resolution because it includes signal properties into the algorithm for estimating source activity. In contrast, dipole modeling is based on the physical sensitivity of the MEG sensors only. The synthetic aperture magnetometry (SAM) minimum-variance beamformer algorithm [88] was used as a spatial filter to estimate the source activity on a lattice of 8 mm spacing across the whole brain volume. Waveforms of averaged source activity across all trials for each stimulus type were calculated following the event-related SAM (ER-SAM) approach [89]. The beamformer analysis using the algorithm as implemented in the VSM software package was based on individual multi-sphere models, for which single spheres were locally approximated for each of the 151 MEG sensors to the three-dimensionally digitized head shape. The ER-SAM procedure results in a z-score of the source activity, which is normalized to an estimate of the sensor noise [90]. For obtaining better interpretable measures, we normalized the event related source activity with respect to the spontaneous brain activity. Therefore, we calculated the mean variance across all time points within an epoch [91] and normalized the time series of source activity by the variance, which resulted in measures of signal-to-noise ratios (SNR) for each voxel [92]. Volumetric maps of group mean SNR values at selected time points were overlaid with the anatomical image of a template brain (colin27, Montreal Neurological Institute) [93] and were visualized with AFNI software (National Institute of Mental Health, Bethesda, MD, USA) [94].

### Multivariate analysis of source activity

Significant contrasts in spatial-temporal patterns of SAM source activities for the three recording sessions and the two speech stimuli were examined by multivariate partial least squares (PLS) analysis [42,95]. The main question was, whether a spatio-temporal pattern of source activity exists as a factor that explains a difference in the representation of the two speech stimuli after training. As a multivariate technique similar to principal component analysis, the PLS is suitable for identifying the relationship between one set of experimental parameters as independent variables and a large set of dependent measures (i.e. the neuroimaging data). PLS has been successfully applied to time-series of multi-electrode event-related potential [24], functional MRI [96], and MEG [97]. The PLS provides a set of latent variables (LVs), obtained by singular value decomposition of the spatio-temporal measures of source activity. The LVs are ordered according to the amount of covariance of the data matrix they are accounting for. Each LV explaining a specific pattern of experimental conditions is expressed by a cohesive spatial-temporal pattern of brain activity. The significance of each LV was determined by a permutation test for which the conditions were randomly reassigned for 500 re-computations of the PLS, which yielded the empirical distribution for the singular values under the null hypothesis. An LV was considered to be significant at p < 0.05. For each significant LV, the reliability of the corresponding eigenimage of brain activity was assessed by bootstrap estimation using 500 resampled sets of data with the subjects randomly replaced for re-computation of PLS, at each time point at each location.

## Competing interests

The authors declare that they have no competing interest.

## Authors’ contributions

BR and KT conceived and designed the study and performed the data acquisition. BR analyzed the data, SJ developed and performed the source analysis, BR, SJ, and KT drafted the manuscript. All authors read and approved final manuscript.
